# Plasma Expression of Carotid Plaque Presence-Related MicroRNAs Is Associated with Inflammation in Patients with Rheumatoid Arthritis

**DOI:** 10.3390/ijms242015347

**Published:** 2023-10-19

**Authors:** Dídac Llop, Silvia Paredes, Daiana Ibarretxe, Delia Taverner, Núria Plana, Roser Rosales, Lluís Masana, Joan Carles Vallvé

**Affiliations:** 1 Unitat de Recerca de Lípids i Arteriosclerosi, Universitat Rovira i Virgili, 43201 Reus, Catalonia, Spain; 2 Institut d’Investigació Sanitària Pere Virgili (IISPV), 43007 Reus, Catalonia, Spain; 3 Centro de Investigación Biomédica en Red de Diabetes y Enfermedades Metabólicas Asociadas, 28029 Madrid, Spain; 4 Sección de Reumatología, Hospital Universitario Sant Joan, 43204 Reus, Catalonia, Spain; 5 Servicio de Medicina Interna, Hospital Universitario Sant Joan, 43204 Reus, Catalonia, Spain

**Keywords:** rheumatoid arthritis, microRNAs, atherosclerosis, inflammation, biomarker, epigenetics

## Abstract

Rheumatoid arthritis (RA) is associated with problems beyond the joints such as cardiovascular (CV) disease. MicroRNA-24, -146 and -Let7a are associated with carotid plaque presence in RA patients. We evaluated whether these microRNAs were involved in the inflammatory state of RA, and we studied their gene targets to understand their role in inflammation and atherosclerosis. A total of 199 patients with RA were included. Inflammatory variables such as disease activity score 28 (DAS28) and erythrocyte sedimentation rate (ESR) were quantified. MicroRNAs were extracted from plasma and quantified with qPCR. Multivariate models and classification methods were used for analysis. The multivariate models showed that diminished expression of microRNA-146 was associated with inferior levels of DAS28-ESR, and the decreased expression of microRNA-24, -146 and -Let7a were associated with lowered ESR in the overall cohort. When microRNAs were evaluated globally, a global increase was associated with increased DAS28-ESR and ESR in the overall cohort. Sex-stratified analyses showed different associations of these microRNAs with the inflammatory variables. Finally, random forest models showed that microRNAs have a pivotal role in classifying patients with high and low inflammation. Plasmatic expressions of microRNA-24, -146 and -Let7a were associated with inflammatory markers of RA. These microRNAs are associated with both inflammation and atherosclerosis and are potential therapeutic targets for RA.

## 1. Introduction

Rheumatoid arthritis (RA) is a chronic autoimmune disease characterized by a systemic inflammatory state that mainly affects the joint synovium. However, the systemic disturbance characteristic of the disease also causes problems beyond the joints, such as cardiovascular (CV) disease, which is associated with increased morbidity and mortality. It is estimated that the risk of developing CV disease in RA patients is approximately 50% higher than that in the general population [1,2,3]. This increased risk is partly explained by the systemic inflammatory state caused by RA, which leads to an accelerated arteriosclerotic process [4]. For this reason, RA is considered an independent risk factor for developing CV disease [5,6]. The formation of atherosclerotic plaques is the main mechanism in the development of CV disease in patients with RA [7]. CV events occur as a result of rupture of atherosclerotic plaques and consequential thrombosis, which in turn causes vessel occlusion. Furthermore, several longitudinal studies confirmed that the assessment of carotid plaque presence (cPP) is a useful tool to establish CV risk in the general population and in patients with RA [8,9]. The pathogenesis of cPP is multifactorial and includes vascular, metabolic and inflammatory components [10]. Inflammation is an important trigger of plaque instability, and many aspects of the pathophysiology of atherosclerosis are mirrored in the inflamed RA synovium [11]. Moreover, important inflammatory mediators in RA such as TNF-α, IL-6, IL-1 or adhesion molecules contribute to endothelial dysfunction, the accumulation of immune cells in plaques and plaque instability [12].

MicroRNAs (miRNAs) are evolutionarily conserved noncoding single-stranded RNA molecules of approximately 18–22 nucleotides that act as posttranscriptional gene regulators [13]. They negatively regulate the translation of target mRNAs by altering their stability either through binding to their 3′ untranslated region (UTR) or—less frequently—to the 5′ UTR or the coding sequence. As a result, miRNAs can directly target mRNAs for degradation in the presence of perfect complementarity or induce translational repression through different mechanisms [14]. They have a promiscuous nature, as each miRNA targets multiple mRNAs, and each mRNA may be targeted by multiple miRNAs. This nature involves them in a vast number of biological processes such as cellular differentiation, metabolism, atherosclerosis and inflammation [15]. Moreover, different studies pointed out the important role of circulating miRNAs as potential biomarkers of vascular disease due to their high stability and reliable detection within biofluids [16]. For this reason, circulating levels of miRNA-499 and miRNA-21 have been targeted as potential novel biomarkers of acute coronary syndrome and acute myocardial infarction, respectively [17,18]. Specifically, in RA, we observed that the decreased expression of miRNA-24, miRNA-146 and miRNA-Let7a was associated with cPP in male patients [19]. However, several circulating miRNAs have also been targeted as diagnostic, disease severity and inflammatory biomarkers of RA. It was observed that miRNA-24, -146 and -125a were increased in patients with RA compared with healthy controls [20]. Furthermore, it was shown that the expression levels of miRNA-22 and miRNA-223 were increased in anticyclic citrullinated peptide (anti-CCP)-positive populations [21,22] and that miRNA-125b was positively correlated with C-reactive protein (CRP), erythrocyte sedimentation rate (ESR) and disease activity score 28 (DAS28) [23]. Additionally, emerging evidence indicates that circulating miRNAs in blood such as miRNA-122 or miRNA-3925 show differential expression not only between healthy patients and RA patients but also between patients with RA and patients with osteoarthritis, systemic lupus erythematous or Graves’ disease, which gives them excellent potential as biomarkers to differentiate patients with RA from other autoimmune diseases [24].

Given that there is a strong relationship between the chronic inflammatory state of RA and arteriosclerosis and that microRNAs appear to be involved in both processes, we explored whether miRNA-24, -146 and -Let7a, which we previously associated with cPP in male patients with RA [19], could be involved in the inflammatory process of RA and evaluated their role as biomarkers of the prognosis and severity of the disease. These miRNAs were evaluated both individually and globally. In addition, we also studied whether these miRNAs were associated with inflammation in a group of healthy controls and in a group of subjects with metabolic disorders, to assess whether these miRNAs are specifically associated with inflammation in RA or are associated with inflammation in general. Finally, we also performed a gene set enrichment analysis (GSEA) of the targeted genes of miRNA-24, -146 and -Let7a to better understand their roles in RA, inflammation and arteriosclerosis.

## 2. Results

### 2.1. General Characteristics of the Cohort

Table 1 shows the general characteristics of the RA cohort included in this study (n = 199), globally and stratified by sex. The mean age of the cohort was 57.8 ± 12.4 years, the median of the disease duration was 8 (3–13) and 66% of the patients were female. Regarding DAS28-ESR, 56.28% of the patients were in the DAS28 ≥ 3.2 group. Significantly more women were in the DAS28 ≥ 3.2 group. Moreover, women showed increased DAS28-CRP, number of swollen joints and clinical disease activity index (CDAI). A total of 63.81% of the patients were in the pathological-ESR (PAT-ESR) group. Positive rheumatoid factor (RF) was detected in 74.37% of the patients, 73.86% were positive for anti-CCP and 57.77% presented erosions. A total of 74.8% received conventional synthetic disease-modifying antirheumatic drugs (csDMARDs), 21.6% received biological treatment, 57.28% received nonsteroidal anti-inflammatory drugs (NSAIDs) and 51% received glucocorticoids (mean dose 2.91 mg orally/day). Waist circumference, systolic blood pressure (SBP) and diastolic blood pressure (DBP) were increased and HDLc was decreased in men. Finally, more male patients presented hypertension than female patients. Supplementary Table S1 shows the general characteristics of the RA cohort, control subjects (n = 64) and patients with metabolic disorders (n = 82). Overall, similar variable values are observed between RA and metabolic patients, which are increased compared to controls. In addition, RA patients have increased ESR, CRP and fibrinogen compared to metabolic subjects.

### 2.2. Associations of miRNAs with Inflammatory Indexes

**Figure 1:**
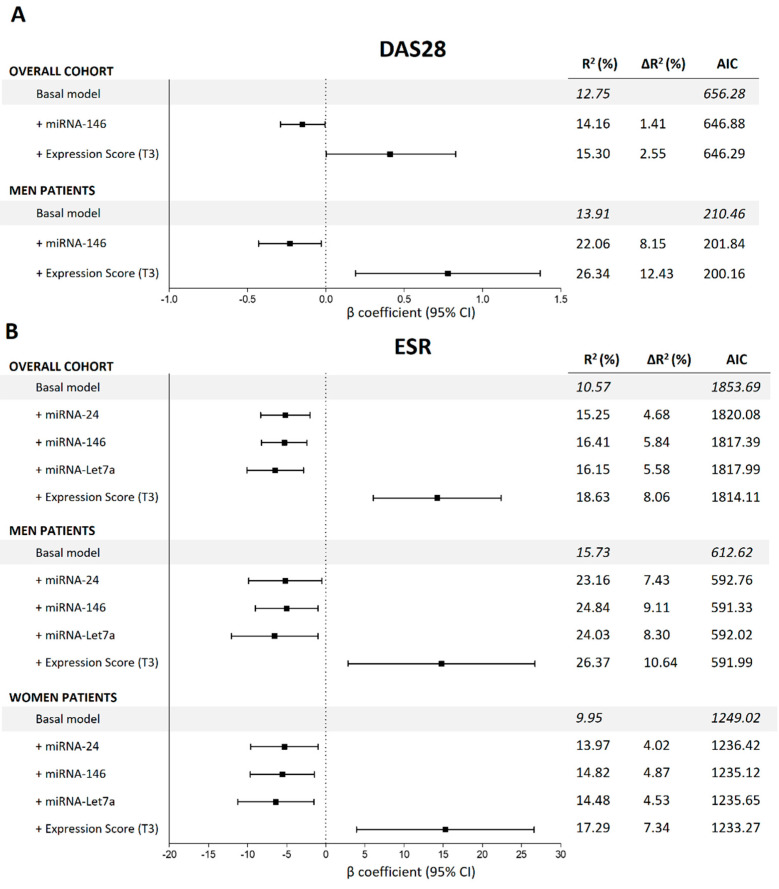
Summaries of the multivariate lineal regression models to estimate the associations between miRNAs and DAS28-ESR and ESR in the overall cohort and stratified by sex. All the basal models are adjusted for age, sex, body mass index, disease duration, disease-modifying antirheumatic drugs, nonsteroidal anti-inflammatory drugs, corticosteroids and biological drugs. (**A**) miRNA associations with DAS28-ESR; (**B**) miRNA associations with ESR. DAS28-ESR = disease activity score 28, ESR = erythrocyte sedimentation rate, β = beta coefficient, AIC = Akaike information criteria, T3 = 3rd tertile.

Figure 1 shows the different β coefficients of the miRNAs statistically associated with DAS28-ESR and ESR along with their respective confidence intervals (CI). First, in the overall cohort, multivariate linear models adjusted for multiple confounders (Figure 1 legend) showed that the decreased expression of miRNA-146 (β = −0.15) was significantly associated with lowered DAS28-ESR. Moreover, reduced expressions of miRNA-24 (β = −5.17), miRNA-146 (β = −5.29) and miRNA-Let7a (β = −6.47) were significantly associated with inferior levels of ESR. Second, we observed that the decreased expression of miRNA-146 was associated with DAS28-ESR in men but not in women. Finally, reduced expression levels of miRNA-24, -146 and -Let7a were associated with decreased ESR in both men and women.

We then evaluated the global quality of the models in terms of increased variability explained (∆R^2^) and Akaike information criteria (AIC) (Figure 1). The addition of the different miRNAs to the basal model significantly increased the ∆R^2^ of DAS28-ESR and ESR and dropped the AIC, showing an improvement in the global quality of the models. The complete summary of the models is shown in Figure 1.

No statistically significant associations were observed between the selected miRNAs and DAS28-CRP or CDAI.

### 2.3. Associations of miRNAs with DAS28-CAT and PAT-ESR

**Figure 2:**
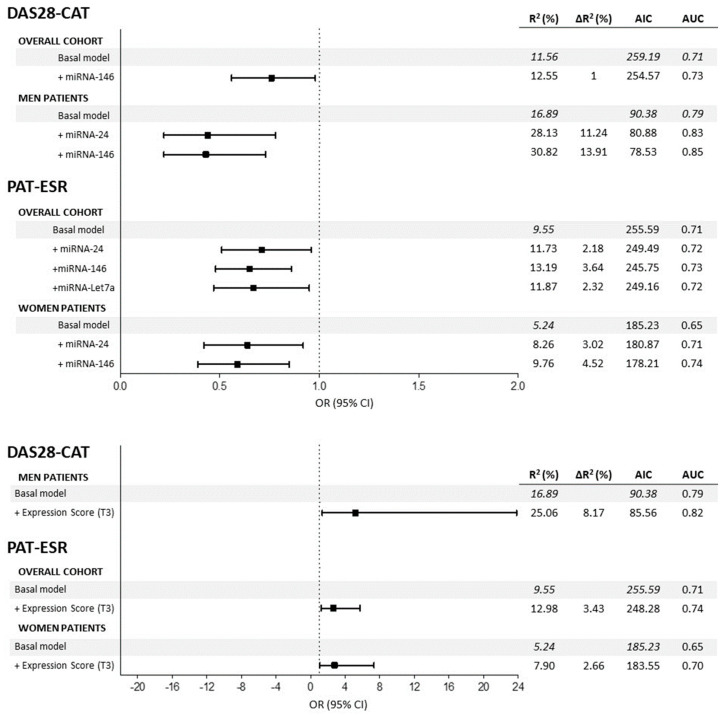
Summaries of the multivariate logistic regression models to estimate the associations between miRNAs and DAS28-CAT and PAT-ESR in the overall cohort and stratified by sex. All the basal models are adjusted for age, sex, body mass index, disease duration, disease-modifying antirheumatic drugs, nonsteroidal anti-inflammatory drugs, corticosteroids and biological drugs. DAS28 = disease activity score 28, ESR = erythrocyte sedimentation rate, β = beta coefficient, AUC = area under the curve, AIC = Akaike information criteria, T3 = 3rd tertile.

**Figure 3:**
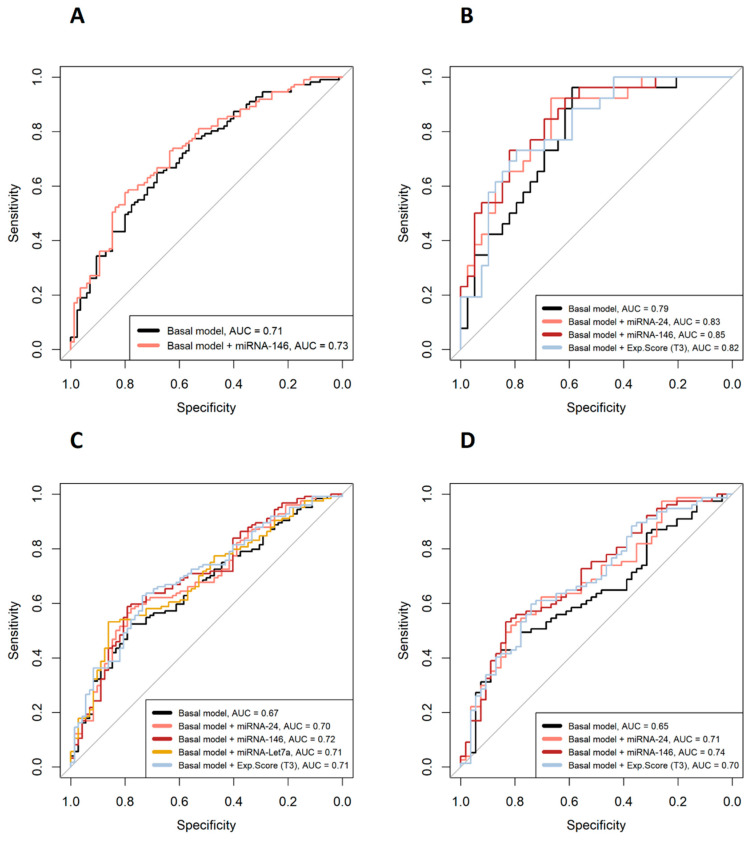
ROC curves of the multivariate logistic models. (**A**) DAS28 > 3.2 or DAS28 ≤ 3.2 patients in the overall cohort; (**B**) DAS28 > 3.2 or DAS28 ≤ 3.2 in male patients; (**C**) PAT-ESR in the overall cohort; (**D**) PAT-ESR in women. Basal models are adjusted for age, sex, BMI, disease duration, csDMARDs, NSDAIDs, biological drugs and glucocorticoids. AUC = area under the curve.

**Figure 4:**
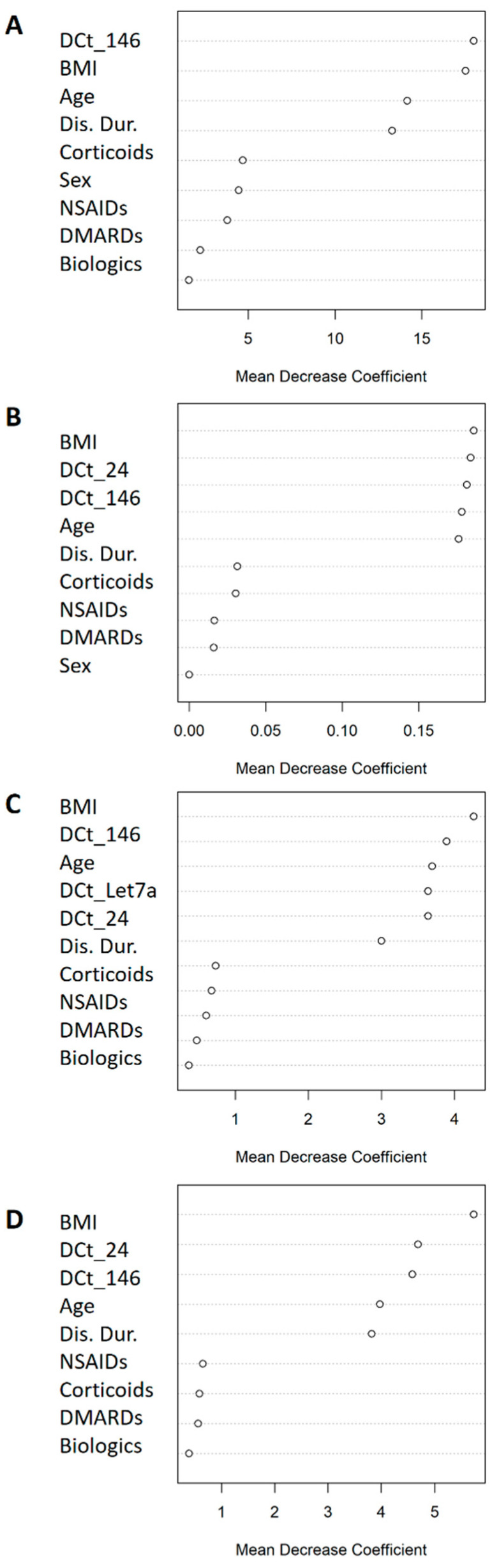
Mean decrease coefficient variable importance classifying DAS28 > 3.2/ DAS28 ≤ 3.2 patients and PAT-ESR. (**A**) DAS28 > 3.2/DAS28 ≤ 3.2 patients in the overall cohort; (**B**) DAS28 > 3.2/DAS28 ≤ 3.2 in male patients; (**C**) ESR-PAT in the overall cohort; (**D**) ESR-PAT in women. All the models were adjusted for the statistically significant miRNAs and the confounder variables.

Figure 2 shows the odds ratios (OR) of the miRNAs associated with categorical clinical variables along with their confidence intervals. First, in the overall cohort, multivariate logistic models adjusted for multiple confounders (Figure 2 legend) showed that reduced expression of miRNA-146 (OR = 0.76) decreased the odds of being in the DAS28 > 3.2 group (based on DAS28-CAT). Regarding PAT-ESR, inferior levels of miRNA-24 (OR = 0.70), -146 (OR = 0.65) and -Let7a (OR = 0.66) minimized the odds of being in the pathological group. Second, when the analyses were stratified by sex, male patients who showed a decrease in the expressions of miRNA-24 (OR = 0.44) and miRNA-146 (OR = 0.43) had lowered odds of being in the DAS28 > 3.2 group. Finally, in women, decreased expressions of miRNA-24 (OR = 0.64) and miRNA-146 (OR = 0.59) significantly reduced the odds of being in the PAT-ESR group. The amount of variability explained increased (R^2^ and ∆R^2^) and the AIC dropped when the different miRNAs were added into the models, improving the quality of the model performance (Figure 2). Furthermore, adding the different miRNAs to the basal model increased the area under the curve (AUC), which implied an improvement in the classification accuracy of the models (Figure 3). Additionally, random forest models were adjusted with all the significant miRNAs and confounders for each categorical variable to evaluate the importance of the miRNAs with an alternative classification method. Figure 4 shows that miRNA-146 in the overall cohort and miRNA-24 and -146 in men play a pivotal role in properly classifying patients into the DAS28 > 3.2 group. Finally, miRNA-24, -146 and -Let7a play pivotal roles in classifying patients into the PAT-ESR group in the overall cohort, as do miRNA-24 and -146 in women. When we studied whether these miRNAs were associated with inflammation in healthy subjects and in patients with metabolic disorders, we observed that neither miRNA-24, -146 nor -Let7a were associated with ESR or C-reactive protein in either group (Supplementary Table S2).

### 2.4. Associations of Global miRNA Expression Scores with Inflammatory Variables

Finally, we analysed the effect of the global expression score (GES) on the different variables studied. Complete summaries are shown in Figure 1 for continuous variables and Figure 2 for categorical variables. First, regarding continuous variables, adjusted models showed that patients with high GES presented significantly increased ESR compared with that in patients with low GES in the overall cohort of men and women. The difference between patients with high GES and low GES was on average 14.23 mm/h in the general cohort, 14.79 mm/h in men and 15.26 mm/h in women. Adding the GES to these models significantly enhanced their quality, achieving an improvement in the explained variability (∆R^2^) of 8.06% in the overall cohort, 10.64% in men and 7.34% in women, and dropping the AIC in all models. Second, we also observed that patients with high GES presented on average 0.41 more points in DAS28-ESR than did patients with low GES in the overall cohort (∆R^2^ = 2.55%) and 0.78 points in male patients (∆R^2^ = 12.43%), improving the global quality of the models (Figure 1). Third, regarding categorical variables, we observed that patients in the overall cohort and women with high GES presented 2.69 and 2.77 augmented odds, respectively, of being in the PAT-ESR group compared with those with low GES. Adding GES to the models improved the ∆R^2^ and the AUC (the respective ∆R^2^ and AUC increased by 3.43% and 4% in the overall cohort and 3% and 5% in women) and dropped the AIC (from 255.59 to 248.28 in the overall cohort and from 185.23 to 183.55 in women). Finally, we also observed that male patients with high GES had 5.16 times higher odds of being in the DAS28 > 3.2 group compared with patients with low GES. Adding the GES to this model also improved its quality (∆R^2^ and AUC increased by 8.17% and 3%, respectively, and AIC dropped from 90.38 to 85.56) (Figure 2). No significant associations were found between GES and DAS28-CRP or CDAI. On the other hand, GES was not associated with inflammation, measured in terms of ESR and C-reactive protein, either in control subjects or in patients with metabolic disorders.

### 2.5. Gene Set Enrichment Analysis

**Figure 5:**
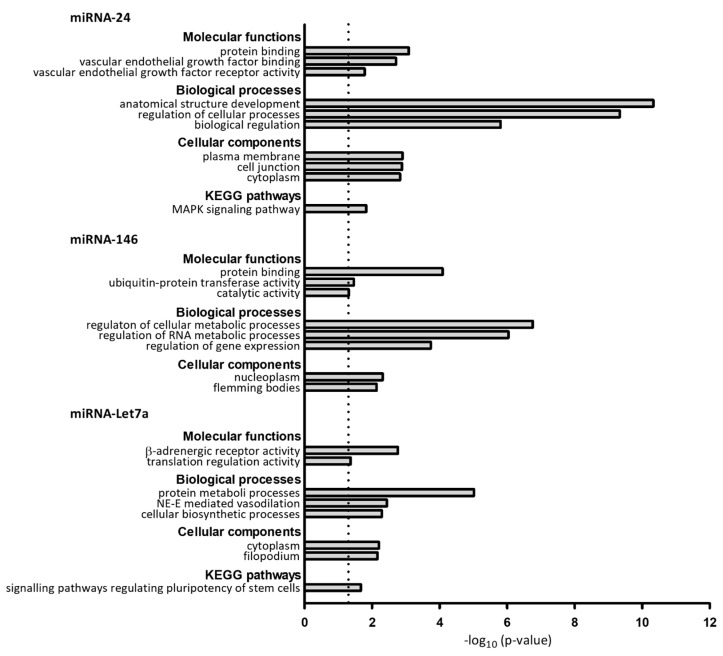
Enrichment analysis of target predicted genes of miRNA-24, miRNA-146 and miRNA-Let7a. Significant biological processes, molecular functions, cellular components and KEGG pathways are showed. NE-E: norepinephrine–epinephrine, KEGG = Kyoto Encyclopedia of Genes and Genomes.

We first predicted possible target genes of miRNA-24, miRNA-146 and -Let7a using the mirDIP v1.4 database. We identified 206 target genes for miRNA-24; 222 for miRNA-146; and 184 for miRNA-Let7a. The gene ontology (GO) functional enrichment analysis showed that miRNA-24-targeted genes were significantly enriched in molecular functions (MF) such as protein binding, vascular endothelial growth factor binding and vascular endothelial growth factor receptor activity. Moreover, miRNA-24-targeted genes were also enriched in biological processes (BP) such as anatomical structure development, regulation of cellular processes and biological regulation. Finally, the KEGG pathway analysis showed that these genes were involved in the *MAPK* signalling pathway. Regarding miRNA-146, the target genes were significantly associated with MF such as protein binding, ubiquitin–protein transferase activity and catalytic activity and BP including regulation of cellular metabolic processes, regulation of RNA metabolic processes and regulation of gene expression. However, these genes were not enriched in any KEGG pathway. Finally, regarding miRNA-Let7a, the targeted genes were involved in MF such as beta-adrenergic receptor activity and translation regulator activity and in BP such as protein metabolic processes, norepinephrine–epinephrine-mediated vasodilation involved in regulation of systemic arterial blood pressure and cellular biosynthetic processes. miRNA-Let7a-targeted genes were also involved in the signalling pathways regulating the pluripotency of stem cells. Complete information from the GSEA is shown in Figure 5.

## 3. Discussion

In the present study, we evaluated the associations between different inflammatory and disease activity parameters and miRNA-24, -146 and -Let7a, which we previously associated with subclinical arteriosclerosis in patients with RA [19].

First, when miRNAs were evaluated individually, we observed that RA patients with decreased expression of miRNA-146 showed decreased DAS28-ESR and decreased odds of being in the DAS28 > 3.2 group. Moreover, decreased expressions of miRNA-24, -146 and -Let7a were associated with decreased ESR and with decreased odds of being in the PAT-ESR group. When we performed sex-stratified analyses, we observed that male patients with decreased expression of miRNA-146 and decreased expression of miRNA-24 and miRNA-146 showed decreased DAS28-ESR and decreased odds of being in the DAS28 > 3.2 group, respectively. Moreover, decreased expressions of miRNA-24, -146 and -Let7a were associated with decreased ESR in both men and women, and decreased expressions of miRNA-24 and -146 decreased the odds of being in the PAT-ESR group in women. Second, we showed that patients with high GES presented increased DAS28-ESR and ESR and showed increased odds of being in the PAT-ESR group compared with patients with low GES. When sex-stratified analyses were performed, these associations were also observed in women. In men, DAS28-ESR and the odds of being in the DAS28 > 3.2 group were increased in the high GES group compared with the low GES group. Nevertheless, no statistically significant associations were detected between the selected miRNAs and DAS28-CRP and CDAI. All these results were independent of the specific medication used by the patients, as cDMARDs, NSAIDs, biologic therapy and corticosteroids were controlled for in all adjusted models. Interestingly, the associations of miRNAs with inflammatory parameters were exclusive to our group of RA patients, as no statistically significant associations were found in either control subjects or in patients with metabolic disorders.

Based on our results, a decrease in the expression of miRNA-24, -146 and -Let7a is associated with a decreased inflammatory state in terms of ESR and DAS28-ESR in patients with RA. In this regard, miRNA-24 levels were described as statistically elevated in patients with RA in different studies targeting this miRNA as a potential diagnostic biomarker [25,26,27]. Moreover, consistent with our results, Murata et al. (2013) showed a positive correlation between miRNA-24 and CRP and DAS28-ESR [25]. miRNA-146 is one of the most studied miRNAs related to RA. For example, a meta-analysis showed that miRNA-146 levels were significantly increased in patients with RA compared with control patients. Moreover, a positive correlation between miRNA-146 levels and ESR was also observed. However, the correlation between miRNA-146 and DAS28-ESR was not statistically significant [28]. Other studies also found that miRNA-146 levels increased in the synovial tissue of RA patients compared with controls, which suggests that miRNA-146 is closely involved in the proinflammatory process of RA [29]. miRNA-Let7a has fewer published results relating it to RA. Circulating miRNA-Let7a levels are increased in RA patients and can be used to distinguish RA patients from other populations. miRNA-Let7a is targeted as a potential diagnostic biomarker for RA, particularly in seronegative patients [30]. Evidence shows that these three miRNAs are closely related to the inflammatory process of RA, and our results confirm and add robustness to the previous data, as our models were adjusted for multiple confounders and we evaluated them both globally and by sex.

It is intriguing that the associations between miRNA-24, -146 and -Let7a and specific inflammatory indexes were exclusive to DAS28-ESR. This observation can be elucidated by several factors. Firstly, our RA patient cohort comprised individuals with established RA who had undergone prolonged medical treatment, resulting in a lower prevalence of swollen and painful joints (Table 1) [31]. The reliance on subjective clinical assessment without objective analytical measurements in CDAI could further explain the absence of associations with this index. Moreover, it is essential to consider the unique characteristics of CRP and ESR as inflammatory markers, as they exhibit variations in sensitivity to disease activity [32]. Thus, in several RA patients, CRP can remain normal even during active disease, reflecting short-term inflammation [33]. In contrast, ESR follows a slower, more persistent pattern, revealing subclinical inflammation. DAS28-ESR captures less controlled disease activity, potentially leading to extra-articular manifestations [34].

Less evidence has been published regarding these miRNAs and CV risk in patients with RA. We showed that a decrease in the expression of miRNA-24, -146 and -Let7a, in addition to being associated with less inflammation, was also associated with lower odds of presenting carotid plaque in men with RA [19]. Furthermore, some results suggest that these miRNAs are implicated in several CV complications. For example, miRNA-24 has been associated with ischaemic heart disease as it is enriched in cardiac endothelial cells and acts as a critical regulator of endothelial cell apoptosis and angiogenesis [35]. Moreover, miRNA-146 is upregulated in patients with heart failure and is also a potential biomarker of left ventricular remodelling after acute myocardial infarction [36,37]. The let-7 family, which includes miRNA-Let7a, was found to have aberrant expression in several CV diseases such as stroke, myocardial infarction, cardiac fibrosis and atherosclerosis [38]. All this evidence suggests that the inflammatory process presented in RA patients and the increased CV risk that they show may not be mediated through classical parameters but through other mediators modulated by genetic factors. Interestingly, different associations were found between male and female patients. Although the exact underlying mechanisms are not fully understood, there are several potential factors that contribute to these sex associations. Hormonal, genetic and epigenetic differences may play a role in disease susceptibility. In addition, differences in lifestyle and in environmental exposures may influence disease development and inflammatory processes, thus contributing to the observed sex associations.

Finally, RA is a highly heterogeneous disease characterized by a wide array of signalling pathways involved in its pathogenesis. GSEA results indicated that the target genes of miRNA-24 are associated with vascular endothelial functions and cellular/biological regulation, whereas those of miRNA-146 are linked to protein activity and gene regulation, and miRNA-Let7a targets are involved in translation and blood pressure regulation. Collectively, these miRNAs predominantly affect signalling pathways, vascular regulation and translation activities. It is important to note that RA is a highly heterogeneous disease characterized by a diverse range of signalling pathways involved in its pathogenesis. Our findings suggest that miRNA-24, -146 and -Let7a may be more closely associated with signalling pathways related to ESR rather than those related to CRP. Specifically, miRNA-146 is known to regulate the NF-kB signalling pathway, a pivotal player in chronic inflammation in RA [39]. In summary, miRNA-24, -146 and -Let7a could potentially influence inflammatory and arteriosclerotic patterns from both a protein and genetic perspective. However, further studies are needed to elucidate the exact mechanisms involved [40].

Our study has several limitations. First, we cannot conclude causality in any of the associations that we found due to the cross-sectional design of our study. Second, the selection of our RA patients was regionally focused, so the results might be population-specific and not generalizable across other populations. For this reason, our results should be validated in other cohorts from different regions. Third, the target and molecular implications of our studied miRNAs should be validated in in vitro and functional studies. Finally, to confirm the clinical relevance of the candidate miRNAs, a follow-up study is needed. However, the robustness of our statistical analyses supports the conclusion that the studied miRNAs have a potential role in predicting inflammation in patients with RA.

In conclusion, our study shows a differential relationship, both individually and globally, between miRNA-24 and -146 and -Let7aand inflammation and disease activity in RA that does not hold true in healthy patients or in patients with metabolic diseases. This work provides evidence of a potential role of the candidate miRNAs as useful epigenetic biomarkers of inflammation and links the inflammatory behaviour of RA with their increased CV risk, which suggests their potential as therapeutic targets.

## 4. Materials and Methods

### 4.1. Patients and Clinical Variables

The RA cohort of the present study was previously described [41,42]. Patients consecutively attending the University Hospital Sant Joan de Reus through external consultations, aged between 18 and 80 years, without concurrent illnesses and with an RA diagnosis based on history, clinical examination, laboratory results and imaging and that fulfilled the classification criteria outlined by the American College of Rheumatology in 1987 were included in this study by our rheumatology team. Patients older than 80 years and younger than 18 years, those with acute intercurrent illnesses and those whose disease diagnosis had been changed were excluded. Patients were recruited between November 2011 and January 2015. On the same day of their medical visit, blood collection was performed. Clinical evaluation of the patients was previously described (Supplementary Data S1) [41]. As a measure of disease activity and inflammation, DAS28 was calculated with the ESR and with the CRP. The DAS28 variable was also categorized (DAS28-CAT) as DAS28 ≤ 3.2 and DAS28 > 3.2 according to the criteria of similar publications [43,44]. These cutoffs distinguish between patients in remission or with low disease activity (DAS28 ≤ 3.2) and those with moderate or high disease activity (DAS28 > 3.2), defining treatment targets. Moreover, the pathological ESR (PAT-ESR) variable is a binary variable created from ESR. The categories of the variable were extracted from Tishkowski and Gupta, 2022 [45] (Supplementary Data S1). Additionally, CDAI was calculated as the sum of swollen and painful joint counts, along with the evaluations provided by both the patients and rheumatologist.

We also included patients who willingly participated and were attending the Vascular Medicine and Metabolism Unit of our hospital due to lipid metabolism disturbances and associated disorders such as obesity, type 2 diabetes and metabolic syndrome. Obesity, type 2 diabetes and metabolic syndrome were diagnosed according to standard clinical criteria [46,47,48]. We also included controls who were selected from the hospital staff as healthy subjects without rheumatoid arthritis, type 2 diabetes, obesity, metabolic syndrome or other illnesses.

This study was approved by the Clinical Research Ethics Committee of our hospital (patients with RA: 11-04-28/4proj5, controls and patients with metabolic disorders: CEIm: 222/2020), and all the participants gave written informed consent. We executed the investigation in accordance with our institution’s guidelines and the Declaration of Helsinki.

### 4.2. Laboratory Measurements

Blood samples were collected from the patients, who had fasted for at least 12 h. Plasma was obtained by whole blood centrifugation at 3000 rpm for 10 min, and plasma samples were stored at −80 °C for analysis. Analytical determinations were performed by enzymatic and conventional methods. Analytical determinations included rheumatoid factor (RF), anti-CCP and inflammatory markers (ESR, CRP and fibrinogen) by conventional methods.

### 4.3. Plasma miRNA Expression

miRNA-24, -146 and -Let7a were studied in independent plasma samples from RA patients. These miRNAs were selected because they were previously associated with carotid plaque presence in patients with RA and showed differential expression levels in patients with RA compared to healthy subjects and patients with other diseases [19,25,28,49,50]. Haemolysis evaluation was performed before RNA extraction with aliquots of 200 µL. Haemolysis was discarded after spectrophotometer analysis at λ = 414 nm, corresponding to oxyhaemoglobin contamination. The complete procedure of the plasma miRNA extraction is detailed in Supplementary Data S2. miRNA-16-5p was chosen as a reference for normalization [51]. The relative expression of each miRNA in each sample was calculated using the variable ΔCt, obtained as Ct miRNA candidate—Ct miRNa-16-5p. An increase in the ΔCt variable of a particular miRNA represented a decrease in the expression of that miRNA.

### 4.4. Statistical Analysis

Mean and standard deviation (SD) is provided for normal variables; median and interquartile range (IQR) for non-normal variables; and percentage and number of individuals for categorical variables. T tests, Mann–Whitney U tests and chi-squared tests were used to evaluate differences between normal, non-normal and categorical variables, respectively. To evaluate miRNA associations with continuous and categorical dependent variables, multivariate linear and logistic models were adjusted. Receiver operating characteristic (ROC) curves and area under the ROC curve (AUC) values were calculated as a measure of the classification accuracy. Random forest (RF) models were also applied with all the significant miRNAs and known confounders for each categorical variable to study the most important classifiers [52]. Details about RF are provided in Supplementary Data S2. All the models were adjusted for age, sex, body mass index (BMI), disease duration and drug consumption and analysed in the overall cohort and stratified by sex. R^2^, ∆R^2^ and Akaike information criteria (AIC) were calculated for each model (Supplementary Data S3). Presenting these parameters provides an insight into the contribution of the different miRNAs to the overall model qualities. We also computed a global miRNA expression score (GES) variable to consider the individual expression each miRNA studied (Supplementary Data S3). GES was analysed as tertiles of expression, the first tertile being the lowest expression score and the third the highest expression one. Statistical analyses were performed in R Studio, version 4.0.1. *p*-values < 0.05 and confidence intervals (CI) that did not include 0 in linear models and 1 in logistic regressions were considered statistically significant.

### 4.5. Gene Set Enrichment Analysis (GSEA)

GSEA of the targeted genes of miRNA-24, -146 and -Let7a was performed. Gene targets were predicted using mirDIP v1.4, which is an integrative database of human miRNA target predictions [53]. We selected the top 1% prediction targets of each miRNA according to the integrative score. Then, we performed a functional enrichment analysis using G:Profiler. Gene ontology (GO) terms and pathways from the Kyoto Encyclopedia of Genes and Genomes (KEGG) database were identified in the target gene list of each miRNA [54]. The GO results are shown according to molecular functions (MF), biological processes (BP) and cellular components (CC) where the targeted genes are implied. KEGG pathways [55] are also presented. Each GO term and KEGG pathway is shown with its negative log_10_ adjusted *p* value. −Log_10_ (*p*-value) > 1.30 were considered significant. 

## Figures and Tables

**Table 1:** Description of the general characteristics, the disease features and the treatments of the overall RA cohort and stratified by sex. n = number of individuals, SBP = systolic blood pressure, DBP = diastolic blood pressure, HAQ = health assessment questionnaire index, anti-CCP = anti-citrullinated cyclic peptide, ESR = erythrocyte sedimentation rate, PAT-ESR= pathological ESR, CRP = C-reactive protein, DAS28 = disease activity score, CDAI = clinical disease activity index, DMARDsc = conventional disease-modifying antirheumatic drugs, NSAIDs = nonsteroidal anti-inflammatory drugs, *p* = *p* value.

	RA (n = 199)	Female (n = 132)	Male (n = 67)	*p*
**Characteristics of the groups**				
Sex—female (%, n)	66%, 132			
Age (years, SD)	57.8 (12.4)	57.3 (12.53)	58.7 (12.17)	0.47
Body mass index (kg/m^2^, IQR)	26.8 (23.2–31.2)	26.5 (22.7–31.6)	27.9 (25.7–30.7)	0.13
Waist circumference (cm, SD)	91.9 (15.1)	88 (15.1)	99.5 (12.2)	<0.001
SBP (mmHg, IQR)	135 (120–150)	133.5 (120–148.5)	139 (128–156.5)	0.04
DBP (mmHg, IQR)	80 (71.5–89)	80 (70.8–88)	85 (75–90)	0.02
LDL cholesterol (mg/dL, IQR)	115 (99–135)	115 (97–135.5)	118 (100–134.5)	0.64
HDL cholesterol (mg/dL, IQR)	66 (53.5–75)	69 (61–80.3)	54 (43–66)	<0.001
Triglycerides (mg/dL, IQR)	92 (69–127.5)	88.50 (65.8–125.3)	94 (75–131)	0.31
Glucose (mg/dL, IQR)	89 (82–99)	88 (81–97)	93 (84–102)	0.07
Current smoker (%, n)	27%, 54	28%, 37	25.37%, 17	0.82
Hypertension (%, n)	59.3%, 118	53%, 70	71.6%, 48	0.01
Diabetes mellitus (%, n)	11.6%, 23	10.6%, 14	13.4%, 9	0.72
Dyslipidaemia (%, n)	40.7%, 81	39.4%, 52	43.3%, 29	0.71
**Disease features**				
Disease onset (years, IQR)	8 (3–13)	8.5 (3–13.25)	6 (2–11.50)	0.33
DAS28-ESR (median, IQR)	3.4 (2.6–4.26)	3.6 (2.77–4.62)	3 (2.41–3.70)	<0.001
DAS28 ≤ 3.2 group (%, n)	43.7%, 87	34.8%, 46	61.2%, 41	<0.001
DAS28 > 3.2 group (%, n)	56.3%, 112	65.2%, 86	38.8%, 26	<0.001
**DAS28-CRP**	2.1 (1.3–3.1)	2.4 (1.6–3.2)	1.5 (1.2–2.4)	<0.001
HAQ (median, IQR)	0.3 (0–0.8)	0.5 (0.2–0.9)	0 (0–0.3)	<0.001
Rheumatoid factor + (%, n)	74.4%, 148	72.7%, 96	77.6%, 52	0.57
Anti-CCP + (%, n)	73.9%, 147	74.2%, 98	73.1%, 49	1
Erosions (%, n)	57.8%, 115	59.8%, 79	53.7%, 36	0.50
ESR (mm/h, IQR)	31 (18.5–50.5)	31 (18.8–54)	29 (18.5–46.5)	0.30
PAT-ESR	63.8%, 127	69.6%, 78	73.1%, 49	0.07
CRP (mg/dL, IQR)	0.5 (0.2–0.9)	0.4 (0.2–0.9)	0.4 (0.2–0.1)	0.68
Fibrinogen (mg/dL, SD)	445.6 (96.5)	442.2 (95.5)	452.4 (98.9)	0.49
**Swollen joints**	1 (0–2)	1 (0–2)	0 (0–2)	0.07
**Painful joints**	1 (0–3)	2 (0–4)	0 (0–2)	<0.001
**CDAI**	6 (2–12)	8 (3.8–15)	3 (0–8)	<0.001
**Treatments (%, n)**				
DMARDsc	74.8%, 149	71.2%, 94	82.1%, 55	0.13
Biological agent	21.6%, 43	24.2%, 32	16.4%, 11	0.27
NSAIDs	57.3%, 114	58.3%, 77	55.2%, 37	0.79
**Glucocorticoids**				
(mean dose: 2.91 mg orally/day)	51%, 102	52.3%, 69	49.3%, 33	0.80

## Data Availability

The data presented in this study are available on request from the corresponding author. The data are not publicly available due to privacy conditions.

## Supplementary Materials

The following supporting information can be downloaded at: https://www.mdpi.com/article/10.3390/ijms242015347/s1.
