# Supplementary material for: Plasma Expression of Carotid Plaque Presence-Related MicroRNAs Is Associated with Inflammation in Patients with Rheumatoid Arthritis
Source: Int J Mol Sci. 2023 Oct 19;24(20):15347. doi: 10.3390/ijms242015347 (PMC10607586; doi:10.3390/ijms242015347)
Supplement: Supplementary file 1 [file ijms-24-15347-s001.zip › Supplementary Methods.pdf]

Supplementary Methods

Supplementary Data S1:

Clinical evaluation: Information on the presence of classical CV risk factors (smoking, hypertension, diabetes and dyslipidaemia), as well as the history of CV events and drug consumption, was collected. Clinical measures such as body weight, height, body mass index (BMI), waist circumference (WC), systolic blood pressure (SBP) and diastolic blood pressure (DBP) were assessed. RF positivity (RF+) was defined as RF values>20, and anti-CCP positivity (anti-CCP+) was defined as anti-CCP values > 3. Dyslipidaemia was defined as having HDLc (High density lipoprotein cholesterol) < 50 mg/dL for women or < 40 mg/dL for men or TG (triglycerides) > 150 mg/dL or LDLc (Low density lipoprotein cholesterol) > 100 mg/dL or treatment with hypocholesterolaemic drugs.

PAT-ESR: Men<50 years old and with ESR>15, men>50 years old with ESR>20, women<50 and with ESR>20 and women>50 and with ESR>30 were considered into the PAT-ESR group.

Supplementary Data S2:

miRNA extraction: 200µl of frozen plasma were used to extract the RNA containing the fraction of small RNAs by means of the miRCURY RNA Isolation Kit (Exiqon) following the manufacturer's instructions. 1µl of a mixture of synthetic RNAs (UniSp2, UniSp4, and UniSp5) was spiked into the plasma to control for the efficiency of the RNA extraction. Additionally, 1.25 µL of MS2 RNA carrier (Roche) was added to improve RNA extraction. MiRNA candidates were measured by qPCR using miRCURY LNA Universal RT microRNA PCR, ExiLENT SYBR Green master mix Kit (Exiqon, Denmark) and primers for each miRNA (hsa-miR LNA™ PCR primer set, UniRT). Melting curve analysis was performed to control the specificity of the qPCR. The cycle threshold (Ct) for each sample and miRNA was obtained with SDS v2.3 software (Applied Biosystems).

Supplementary Data S3:

Statistical analysis:

RF is a supervised classification algorithm based in the growth of conditional inference trees that allows evaluating the importance of each variable in the classification. RF uses bootstrap sampling and feature sampling, which avoids multicollinearity problems. The importance of each variable is measured in terms of mean decrease in Gini coefficient, which measures how each variable contributes to the homogeneity of the nodes and leaves in the resulting RF.

The R-squared (R^2) statistic estimates the amount of variability explained by the model. ΔR^2 shows the increase in variability explained when the different miRNAs were included in the models. Finally, AIC estimates the quality of the model. A lower AIC value implies a better model quality.

The global expression score (GES) was calculated as follows: the expression of each miRNA was divided into tertiles, and we assigned a score of 1, 2 or 3 to the lowest, middle and highest expression tertiles, respectively. The punctuation of the different miRNAs was summed to obtain the GES of each patient.
