# Supplementary material for: Plasma Expression of Carotid Plaque Presence-Related MicroRNAs Is Associated with Inflammation in Patients with Rheumatoid Arthritis
Source: Int J Mol Sci. 2023 Oct 19;24(20):15347. doi: 10.3390/ijms242015347 (PMC10607586; doi:10.3390/ijms242015347)
Supplement: Supplementary file 1 [file ijms-24-15347-s001.zip › Supplementary Tables.pdf]

	**RA** (n = 199)	**Controls** (n = 64)	**Metabolic diseases** (n = 82)	**p**
Sex – female (%, n)	66%, 132	78%, 50	70.73%, 58	0.20
Age (years, SD)	57.8 (12.4)	51.23 (9.12)	62.76 (11.89)	<0.001
Body mass index (kg/m3, IQR)	26.80 (23.24 – 31.19)	24.58 (21.65 – 27.32)	26.56 (23.34 – 29.69)	<0.001
Waist circumference (cm, SD)	91.88 (15.11)	86.28 (10.31)	95.55 (13.92)	<0.001
SBP (mmHg, IQR)	135 (120 – 150)	120 (110 – 130)	135 (123 – 149.8)	<0.001
DBP (mmHg, SD)	81.17 (12.42)	72.50 (9.70)	80 (10.33)	<0.001
LDL cholesterol (mg/dL, SD)	118.6 (31.73)	114.4 (22.12)	124 (31.52)	0.15
HDL cholesterol (mg/dL, IQR)	66 (53.50 – 75)	69 (59.75 – 77.25)	62 (49.5 – 75)	0.17
Tryglycerides (mg/dL, IQR)	92 (69 – 127.5)	76 (57 – 93.25)	93.5 (75 – 141.5)	<0.001
Glucose (mg/dL, IQR)	93.87 (22.77)	90.75 (8.37)	97.23 (15)	0.13
Current smoker (%, n)	27%, 54	15.62%, 10	9.75%, 8	0.003
Hypertension (%, n)	59.29%, 118	10.93%, 7	60.97%, 50	<0.001
Diabetes mellitus (%, n)	11.55%, 23	0%, 0	6.10%, 5	0.01
Dyslipidaemia (%, n)	40.70%, 81	0%, 0	63.41%, 52	<0.001
ESR (mm/h, IQR)	31 (18.50 – 50.50)	9.50 (5 – 14.25)	16 (9 – 26.50)	<0.001
CRP (mg/dL, IQR)	0.5 (0.2 – 0.9)	0.19 (0.06 – 0.2)	0.20 (0.07 – 0.25)	<0.001

Fibrinogen (mg/dL, SD)	445.64 (96.53)	313.3 (281 – 332.8)	344.8 (290 – 393)	<0.001

Supplementary Table 1: Description of the general characteristics of the RA cohort, the control subjects and the patients with metabolic disorders. Normally distributed data is expressed in terms of mean and standard deviation (SD). Non-normally distributed data is expressed in terms of median and interquartile range (IQR). Categorical data is expressed in percentages. To evaluate differences between groups, ANOVA was used to compare normally distributed data and Kruskal-wallis was used to compare non-parametric data. Chi square (X^2) test was used to compare categorical data. P-values < 0.05 are considered statistically significant. n=number of individuals, SBP=systolic blood pressure, DBP=diastolic blood pressure, ESR=erythrocyte sedimentation rate, CRP=C-reactive protein, p=p value.

Control group (**n = 64**)		
ESR		
	β	p-value
MiRNA-24	-0.45	0.65
MiRNA-146	-0.44	0.30
MiRNA-Let7a	-1.15	0.10
CRP		
	β	p-value
MiRNA-24	0.051	0.20
MiRNA-146	-0.002	0.9
MiRNA-Let7a	0.01	0.65
Metabolic group (**n = 82**)		
ESR		
	β	p-value
MiRNA-24	-0.78	0.47
MiRNA-146	-1.18	0.14
MiRNA-Let7a	-0.91	0.33
CRP		
	β	p-value
MiRNA-24	0.04	0.052
MiRNA-146	0.007	0.59
MiRNA-Let7a	0.026	0.11

Supplementary Table 2: Summaries of the multivariate lineal regression models to estimate the associations between miRNAs and ESR and CRP in the control group and in metabolic subjects. All the basal models are adjusted for age, sex and body mass index.
ESR = erythrocyte sedimentation rate, CRP = C reactive protein, β = beta coefficient, AUC = area under the curve, AIC = Akaike information Criteria, T3 = 3rd tertile.
